# Supplementary material for: Phosphoproteomic analysis of neoadjuvant breast cancer suggests that increased sensitivity to paclitaxel is driven by CDK4 and filamin A
Source: Nat Commun. 2022 Dec 7;13:7529. doi: 10.1038/s41467-022-35065-z (PMC9729295; doi:10.1038/s41467-022-35065-z)

Supplementary Material

Supplementary figures

Supplementary Figure 1

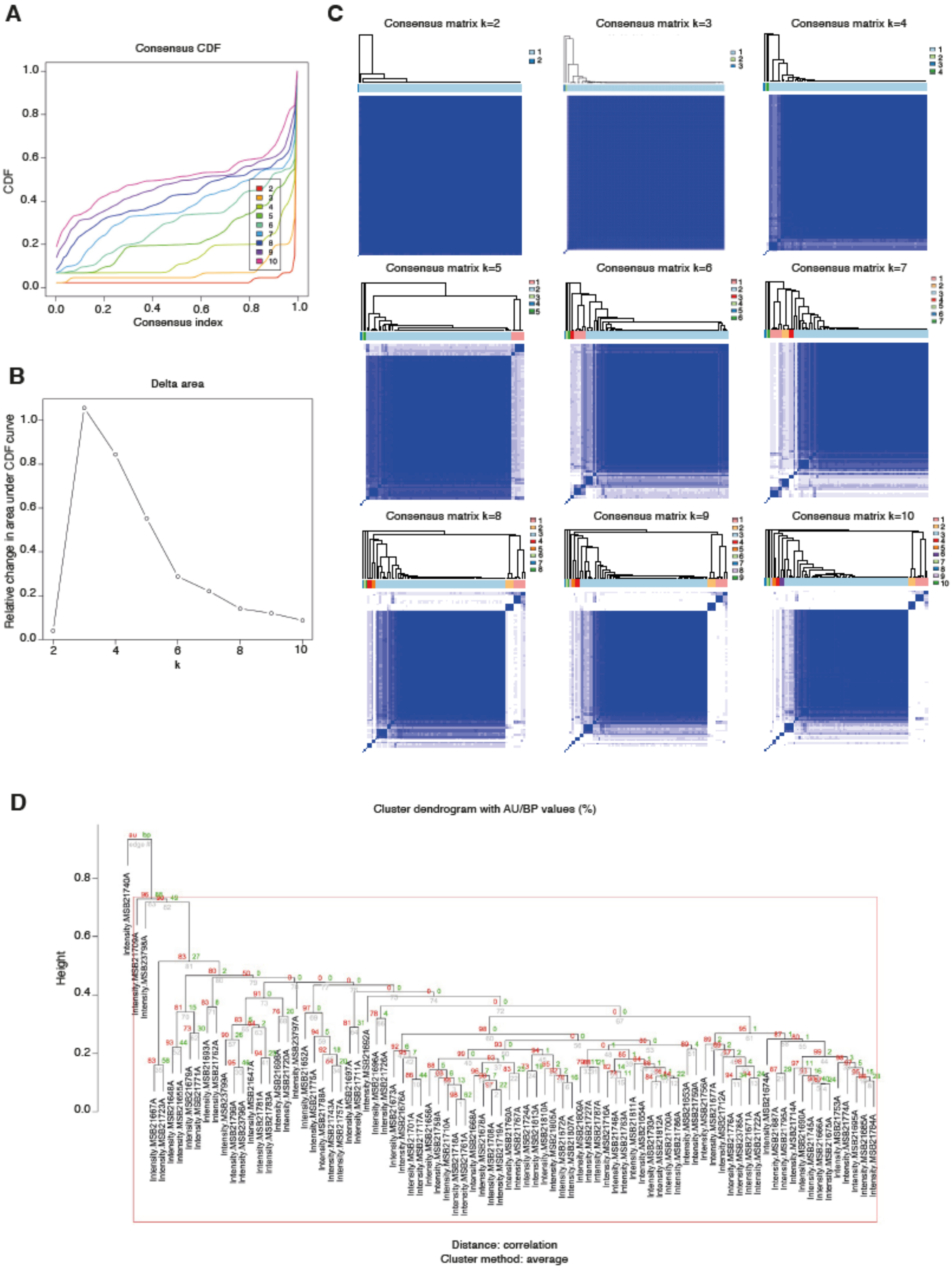

Supplementary Figure 1: Study of samples clustering according to trial arm by consensus clustering and pvclust. We aimed to study further the sample clusters by treatment arm observed in Figure 1C, using two additional methodologies: consensus clustering (A, B and C) and pvclust (D). **(A)** The Cumulative Distribution Function (CDF) curve under different values of k is shown. At optimal k, the area under the CDF curve will not significantly increase with the increase of k value. **(B)** This plot shows the relative change in the area under the CDF curve under different values of k. **(C)** Finally, this panel shows the consensus matrix. Consistency values range from 0 to 1; 0 means never clustering together (white) and 1 means always clustering together (dark blue). Each panel results from increasing k=2 to k=10. According to the consensus CDF **(A)** and delta area **(B)** it could be supported that the optimal number of clusters is k=3; two groups are composed of one sample each, and the third group contains all the remaining samples. We did not observe the 22-sample cluster apparent using Hierarchical Clustering. **(D)** In this panel, the values on the dendrogram correspond to *approximately unbiased* (AU) probability p-values (red, left), *Bootstrap Probability* (BP) values (green, right) and *clusterlabels* (grey, bottom). Clusters with AU >95 are considered to be significant and highlighted in a box. In this case, the pvclust algorithm only found one significant cluster.

Supplementary Figure 2

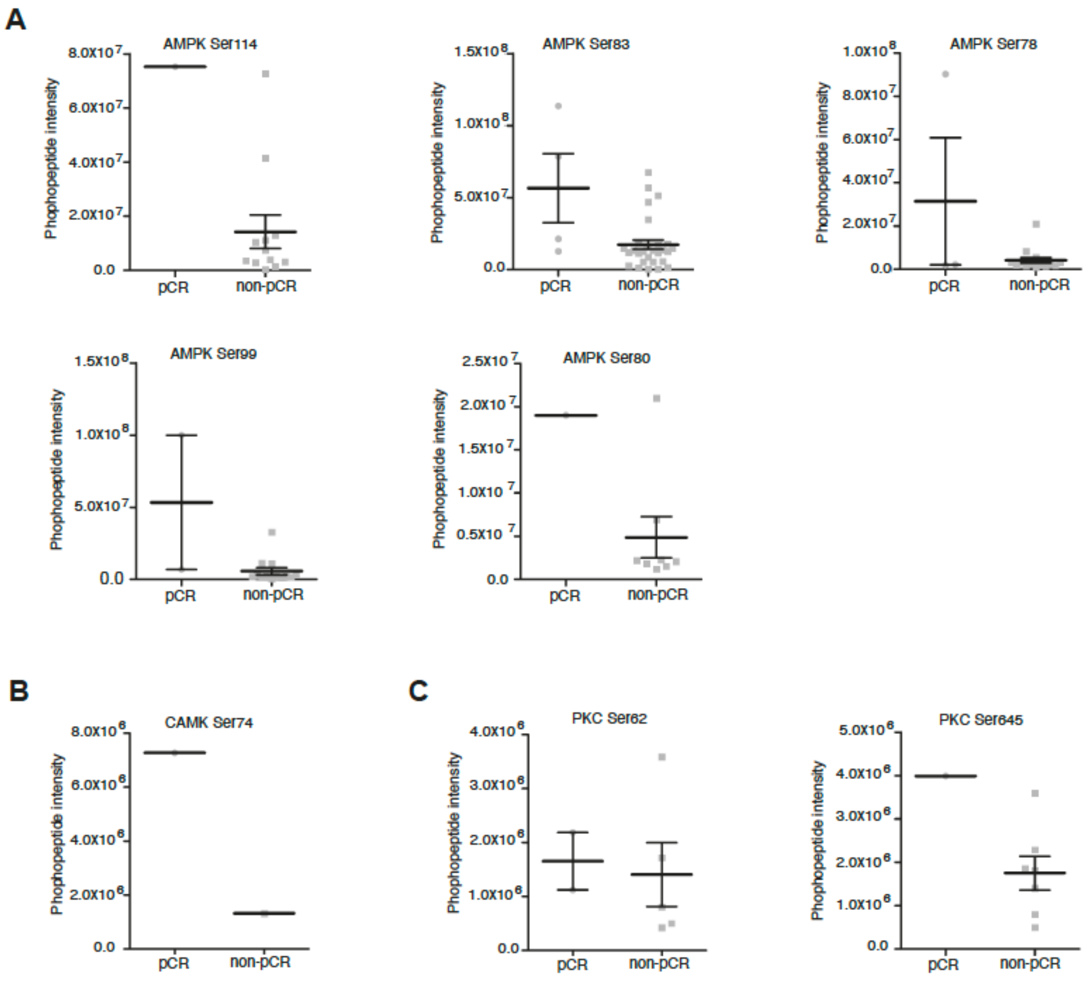

Supplementary Figure 2: Phosphopeptide intensity of peptides mapping to kinases enriched in KSEAS in samples from responders and non-responders. Phosphorylated peptides intensities mapping to **(A)** AMPK, **(B)** CAMK and **(C)** PKC are compared between responder and non-responder patients. Data are presented as mean±SEM. Source data are provided as a Source Data file.

Supplementary Figure 3

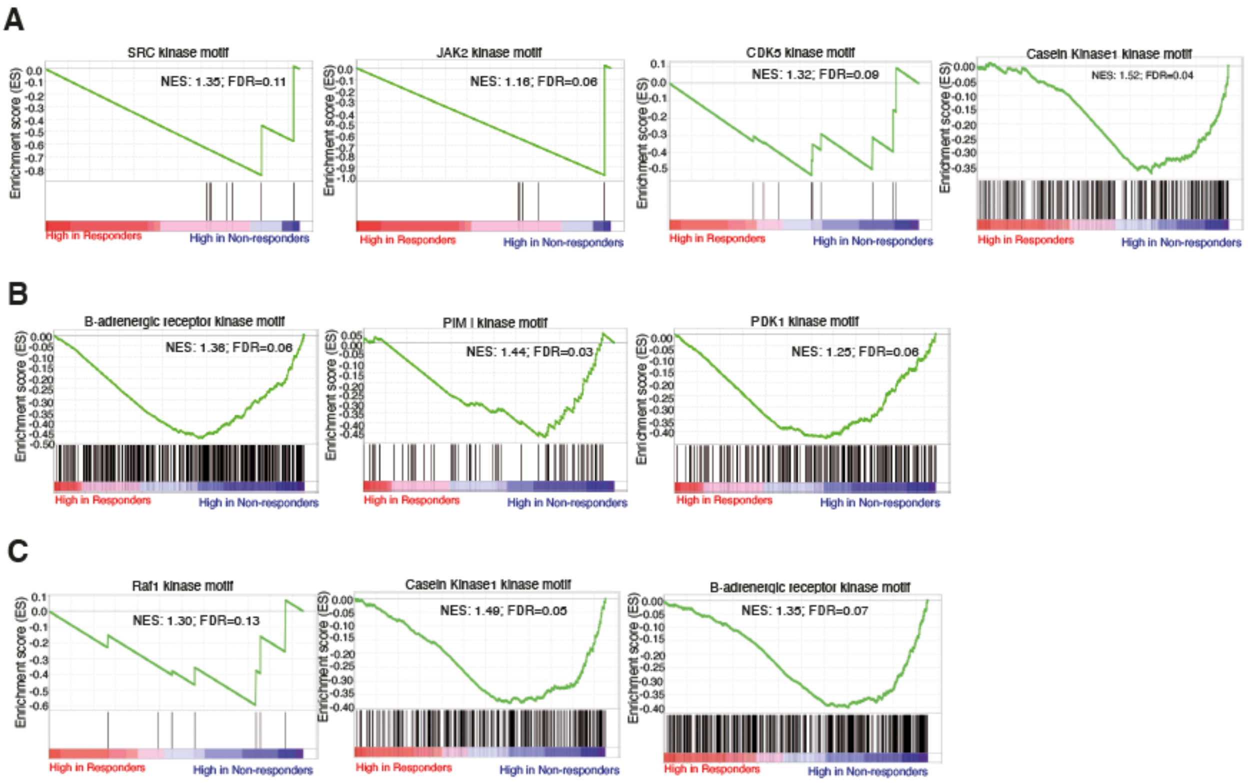

Supplementary Figure 3: Kinases enriched in baseline samples from non-responders to paclitaxel. KSEA plots showing the kinases significantly enriched in the baseline samples of patients that did not achieve pCR considering the whole trial together **(A)**, the experimental arm only **(B)**, or the standard arm only **(C)**. "High in Responders" and "High in Non-responders" refers to the increased abundance of phosphopeptides in the baseline samples of patients that achieved pCR or patients that did not achieve pCR, respectively. NES (Normalized Enrichment Score) and FDR (False Discovery Rate) values are depicted for each KSEA.

Supplementary Figure 4

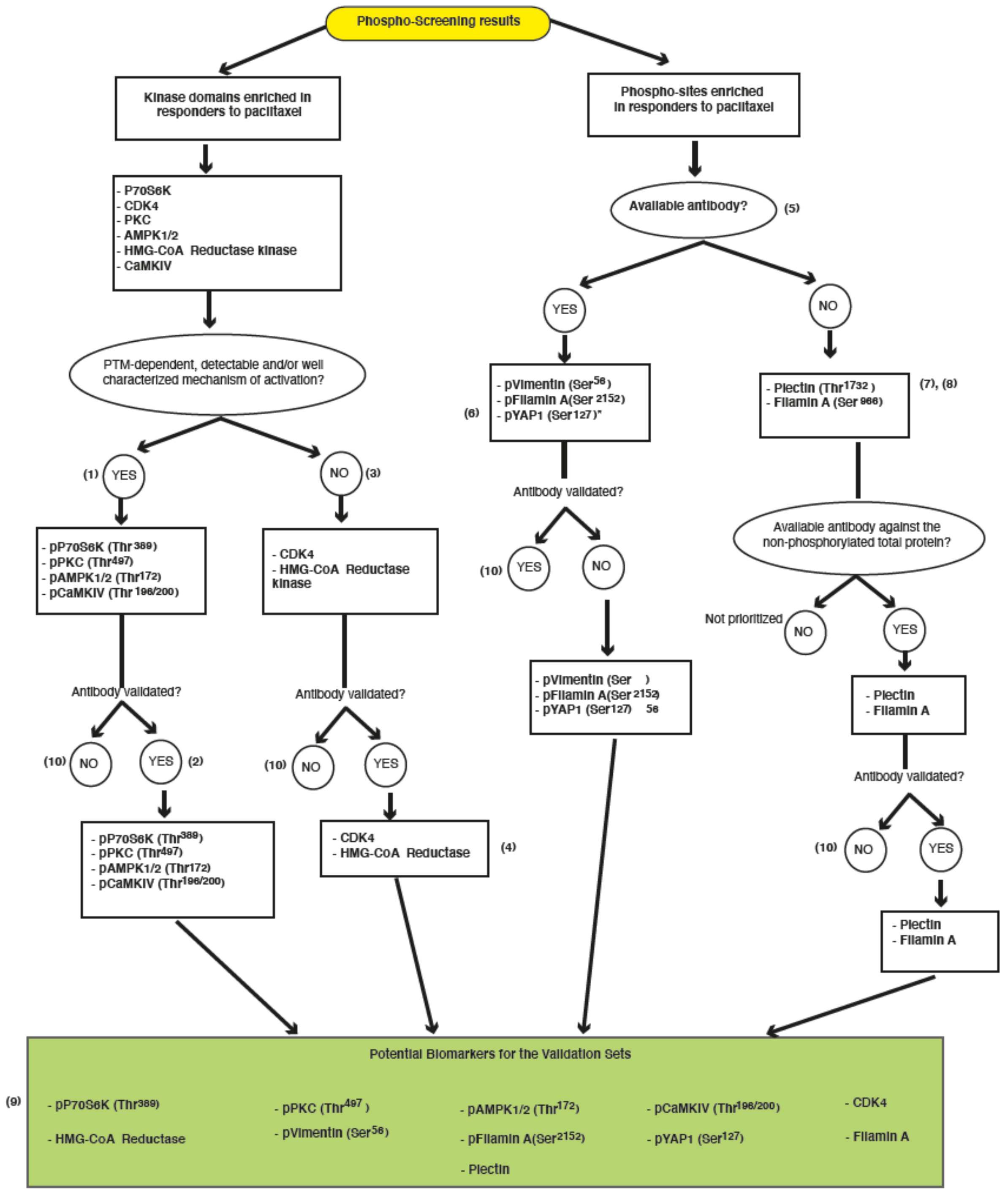

Supplementary Figure 4. Mass spectrometry-to-immunohistochemistry translation algorithm. The algorithm for the selection of antibodies against enriched kinases and phosphorylated proteins identified in the training set specimens, which were subsequently tested in the 2 validation sets, is shown. Translating the information

generated by mass spectrometry assays to biomarkers that can be determined in routine FFPE samples poses two main challenges: 1) unknown criteria for defining an "activated kinase" and identifying the activated form in FFPE samples in many cases; and 2) lack of suitable reagents: currently, antibodies suitable for use in the immunohistochemical detection of several of the kinases or upregulated phosphosites identified by phosphoproteomics are not yet available.

The KSEA approach maps the phospho-sites of a given tumor to one or more kinases able to phosphorylate it. When a given kinase is enriched in a specific phosphoprofile versus one another (i.e., responders versus no responders to paclitaxel), it is suggested that the catalytic activity of that kinase is higher in the former versus the latter tumors. However, the ways by which a specific kinase displays increased activity are quite diverse [transcriptional, post-translational (phosphorylation is the most common, but other modifications are involved in kinase activation), point-mutations, increased ligands or allosteric mechanisms]. Unfortunately, most of these activation mechanisms can not be detected by conventional immunohistochemistry. Thus, detecting such hyper-function in FFPE samples is a complicated task, and requires a case-by-case experimental setup. The following are possible examples: An easy-to-set-up kinase detection case would be P70S6K, where the detection of phosphorylation at p-P70S6K(Thr^{389}), a post-translational modification (PTM) associated with a several-fold increase in catalytic activity[1], in a given sample would imply increased P70S6K activity as compared with a different sample with non-phosphorylated P70S6K. Other kinases are difficult to assess in FFPE samples including those that do not undergo functional modulation based on PTM (CDK4[2]), or a complex, transient, and sometimes incompletely understood chain of events leading to activation (PKC, where different PTMs can induce opposing modulation[3]) or can be activated by changes undetectable by immunohistochemistry (calcium signaling for PKC[4]). Finally, in cases such as HMG-CoA reductase kinase, although the mechanism of activation may be understood[5], we were unable to find suitable immunohistochemistry reagents for its detection and thus we can only indirectly

measure it by determining total levels HMG-CoA reductase. We encountered similar problems for most of the phospho-peptides identified in the volcano plots listed in Table S2, where at best we were able to find antibodies against the total native protein. The depicted algorithm shows the list of steps that we followed to identify the antibodies that should detect, as accurately as possible, the activated kinases or phospho-peptides studied here. For most of the identified domains enriched in responders to paclitaxel, a clear and identifiable mechanism of activation (phosphorylation in all cases) of those kinases was already known **(1)**. The availability of antibodies against the phosphorylated-activated kinase forms allowed direct testing of elevated activity of P70S6K, pan-PKC, AMPK1/2 and CaMKIV **(2)** in the independent validation sets. Regarding the two proteins without a detectable mechanism of activation by immunohistochemistry **(3)**, CDK4 does not undergo post-translational modification but higher transcriptional and protein levels are associated with increased activity; thus, we tested the levels of native CDK4 as a means to stratify validation set patients according to their CDK4 activity[2]. As mentioned, limited by the availability of immunohistochemistry reagents, we chose total HMG-CoA reductase levels as an indirect measure of HMG-CoA reductase kinase activity **(4)**.

Concerning the phospho-sites listed in Table S2, we limited validation to those where, on top of an FDR<0.25, a knowledge-based judgement allowed linking them to some potential pro-survival event in cancer cells or proteins potentially related with the activity of paclitaxel, and just from those proteins where their function is at least somehow characterized **(5)**. For some of them, an antibody against the phosphorylated site was available **(6)**. For the remaining proteins **(7)**, we did not find phospho-specific antibodies and thus we used antibodies against the total protein levels for the validation sets. One of the proteins (Filamin A) was found to be enriched for several phospho-sites **(8)**. This made us suspect a potential relevant implication in sensitivity/resistance to paclitaxel and thus we included both the phosphorylated form (Ser^{2152}) and total Filamin A (as a surrogate for Ser^{1459} and Ser^{996}). The confirmation of positive signal in

immunohistochemistry (Figure S5) let us with 11 potential biomarkers for testing in the external sets **(9)**. Finally, all tested antibodies yielded a signal that was deemed to discriminate successfully between positive and negative cases (shown in Figure S5). Thus, no biomarkers were non-tested because of lack of available antibodies **(10)**. * Only regarding p-YAP(Ser^{109}), we did not find an antibody against Ser^{109} phosphorylation. However, an antibody against Ser^{127} was available and was used instead.

Supplementary Figure 5

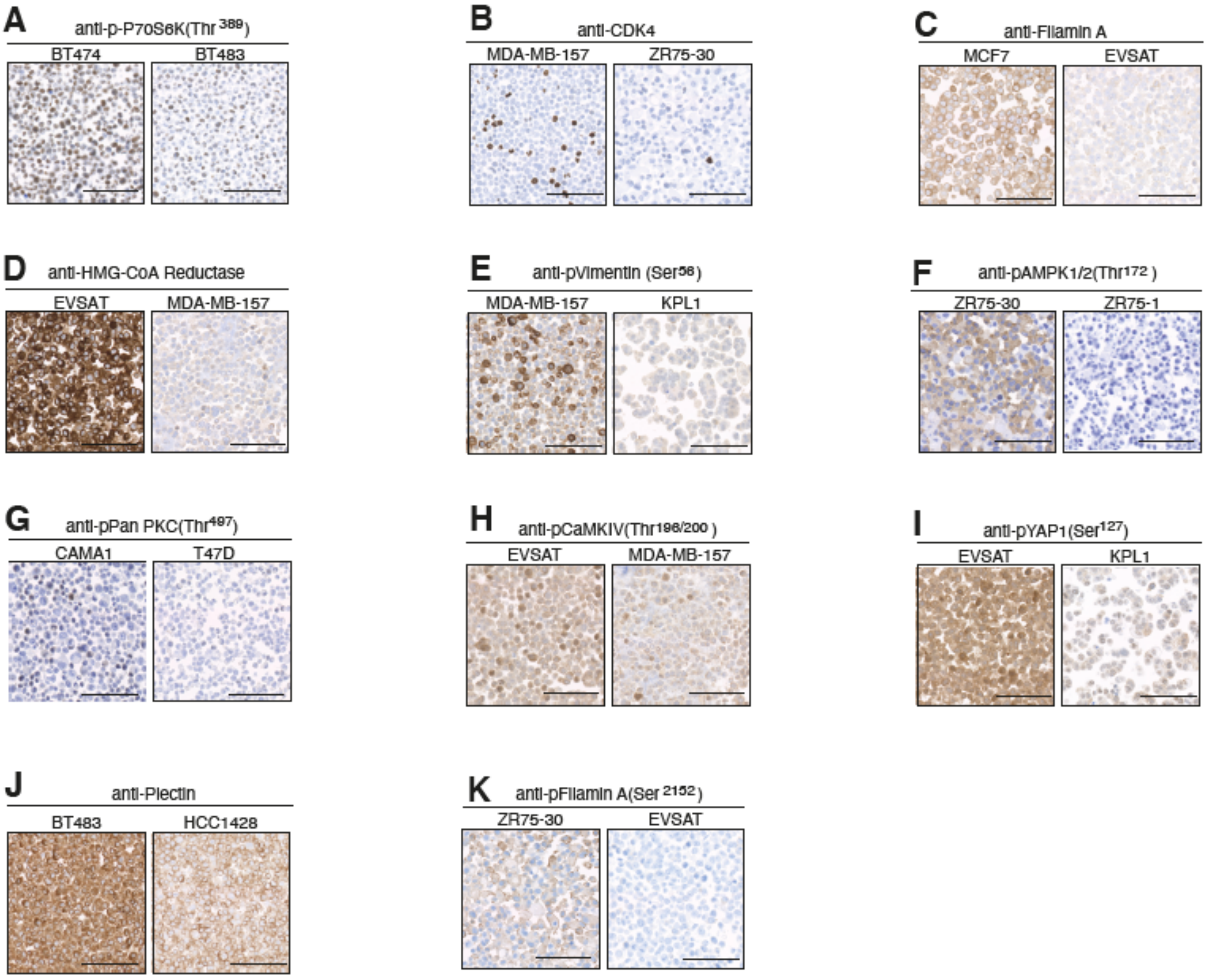

Supplementary Figure 5: Control stainings showing differential immunohistochemical signal in samples with high or low levels of the potential biomarker under study. Breast cancer cell lines were pelleted, fixed with formalin, and embedded in paraffin blocks. Pellet sections were stained following the same protocols as those applied to the TMAs. Each panel **(A to K)** shows representative IHC-stained sections of cell line pellet pairs positive or negative for each of the 11 potential biomarkers defined in Figure S4 subsequently tested in the 2 external sets. Scale bars: 100 micrometers.

Supplementary Figure 6

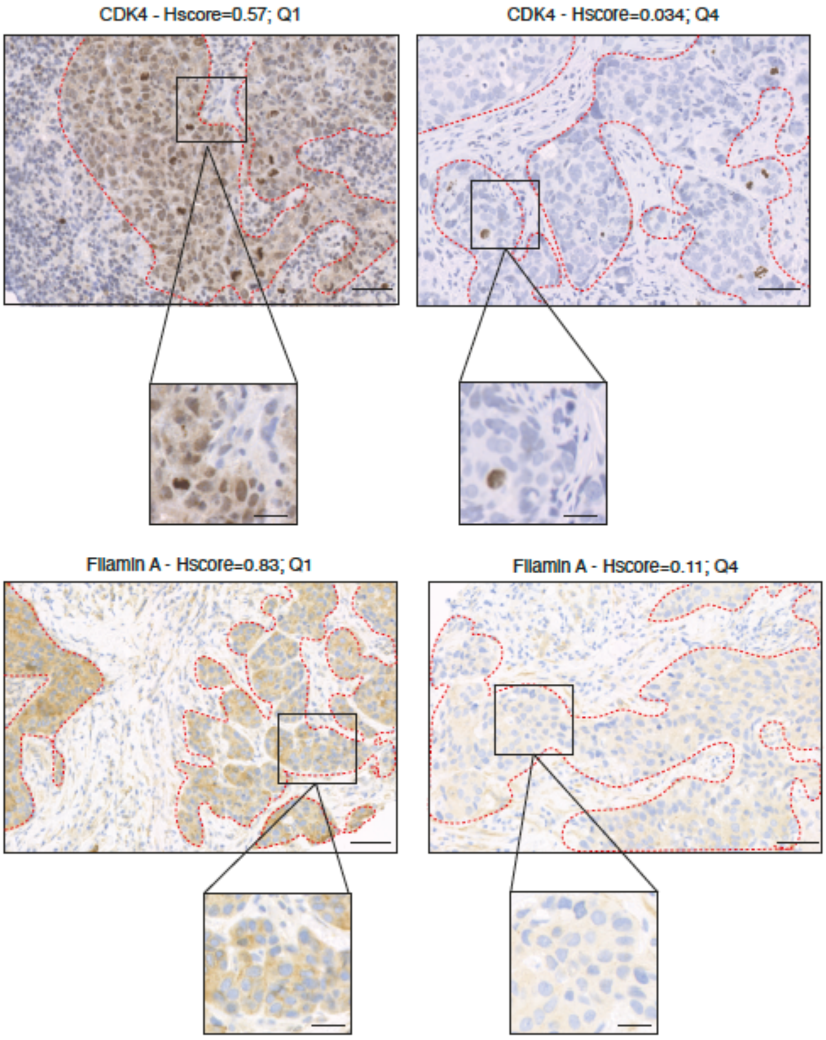

Supplementary Figure 6: Lack of tumor/stromal and/or nuclear/cytoplasmic staining heterogeneity. Examples of patients with intense staining differences are provided. The main source of inter-patient heterogeneity was tumor cell staining, for both markers. The upper panels represent CDK4 staining. Tissue areas framed by the red dashed line correspond to tumor, whereas the rest of the preparation correspond to stromal tissue. Isolated cells with positive nuclear CDK4 were ocassionally observed (left-hand side panel) in some cases, although it was not sufficient to warrant a sub-analysis. Differences in tumor staining are quantified by the computer-aided H-score

calculation; in the case of tumoral CDK4, the H-score varied 15-fold among these two patients. At the cellular level, the staining was observed both in the nuclei and cytoplasm (insets); we did not find patients with only-nuclear or only-cytoplasmic staining. Regarding stromal staining, in general the staining levels were low and similar in all cases; nevertheless, the fact that Set 1 and Set 2 cases were mounted in TMAs precludes a thorough assessment of tumor stroma, since tumor-rich areas were selected. In the lower panels, similar examples are provided for Filamin A. We only observed cytoplasmic staining in all cases. Scale bars: 50 micrometers (insets: 20 micrometers).

Supplementary Figure 7

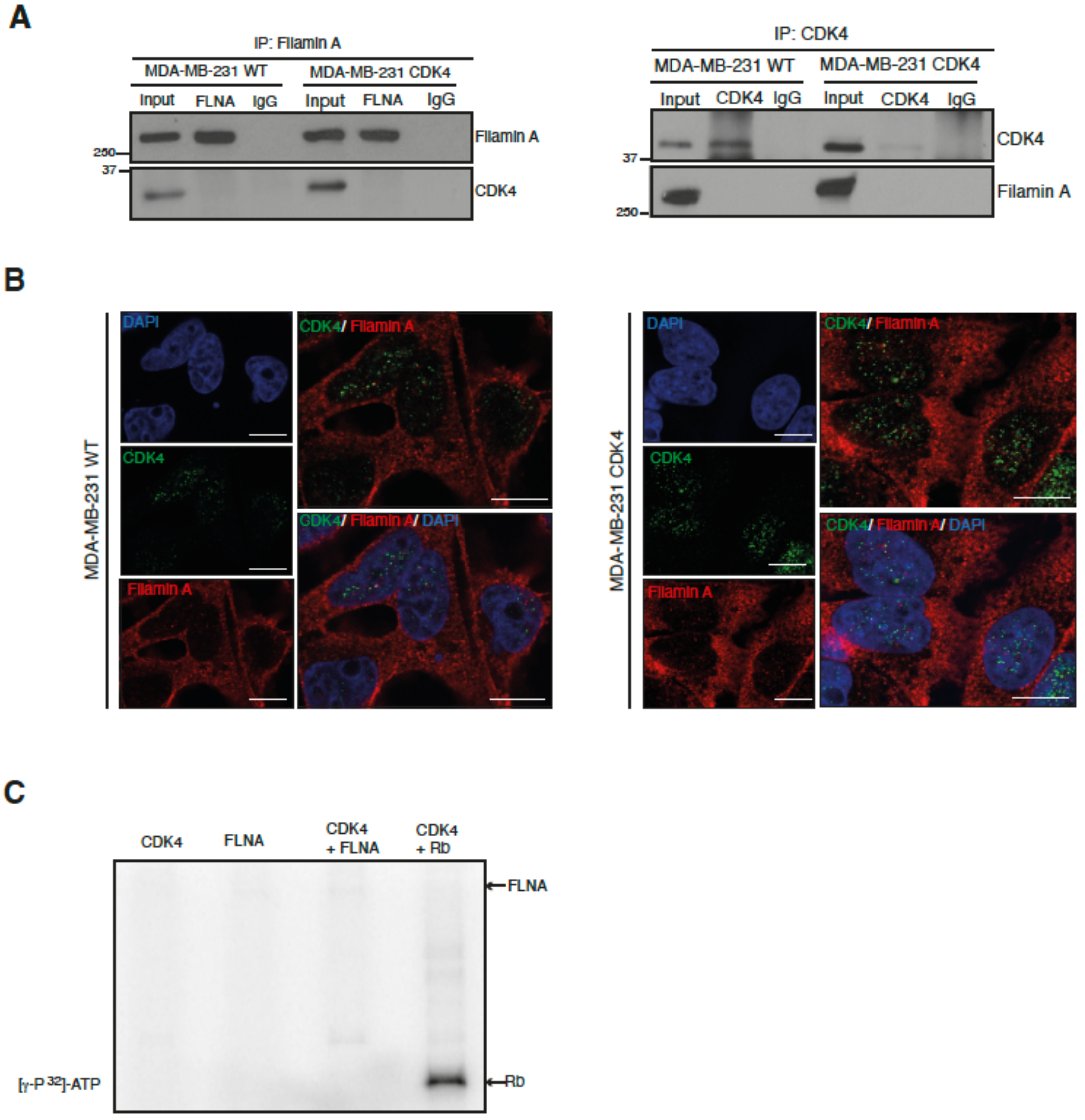

Supplementary Figure 7: Lack of direct interaction between CDK4 and Filamin A. (A) Co-immunoprecipitation assays: in the left-hand side of the figure, the upper panel shows the result of immunoprecipitating Filamin A in parental and MDA-MB-231 CDK4 cell lines. The upper electrophoresis depicts the detection of Filamin A in either whole cell extract ("Input"), immunoprecipitation with anti-Filamin A antibody ("FLNA") and immunoprecipitation with an isotype control antibody (IgG). The lower protein electrophoresis shows that whereas high levels of CDK4 were detectable in the whole cell lysates, no CDK4 was isolated from the Filamin A precipitates in either cell line. In

the right-hand side, the reverse assay is shown: CDK4 immunoprecipitation did not “pull-down” any Filamin A. Samples derive from the same experiment and blots were processed in parallel. **(B)** Immunofluorescence staining of CDK4 and Filamin A acquired by confocal microscopy revealed no evidence of physical co-localization of both proteins in neither cell line, with Filamin A mostly located in the cytoplasm and CDK4 puncta mostly located in the nuclei (Pearson’s co-localization coefficient for both proteins: -0.20 and -0.06 in each cell line) (MDA-MB-231 WT, n=271 cells; MDA-MB-231 CDK4, n=289 cells). Scalebars: 10 micrometers. **(C)** *In vitro* kinase assay testing the activity of CDK4 over Filamin A. As it can be appreciated, CDK4 phosphorylates *in vitro* its known putative substrate Rb, but no radioactive phosphorus is incorporated in the Filamin A lane. Source data are provided as a Source Data file.

Supplementary Figure 8

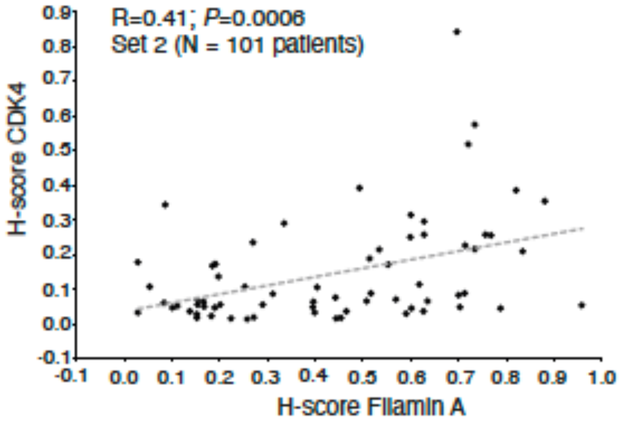

Supplementary Figure 8: Correlation between Filamin A and CDK4 levels in patients. Each dot represents a patient with regard to her Filamin A H-score (X-axis) and CDK4 H-score (Y-axis). A positive correlation was detected between both proteins using a Pearson's correlation coefficient. Source data are provided as a Source Data file.

Supplementary Figure 9

A

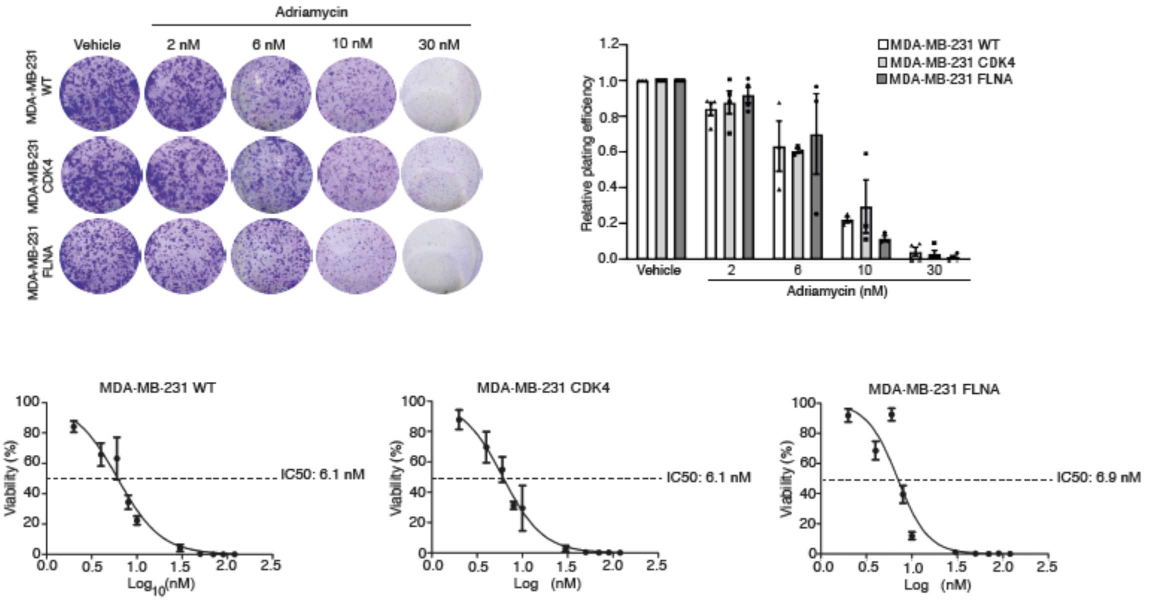

B

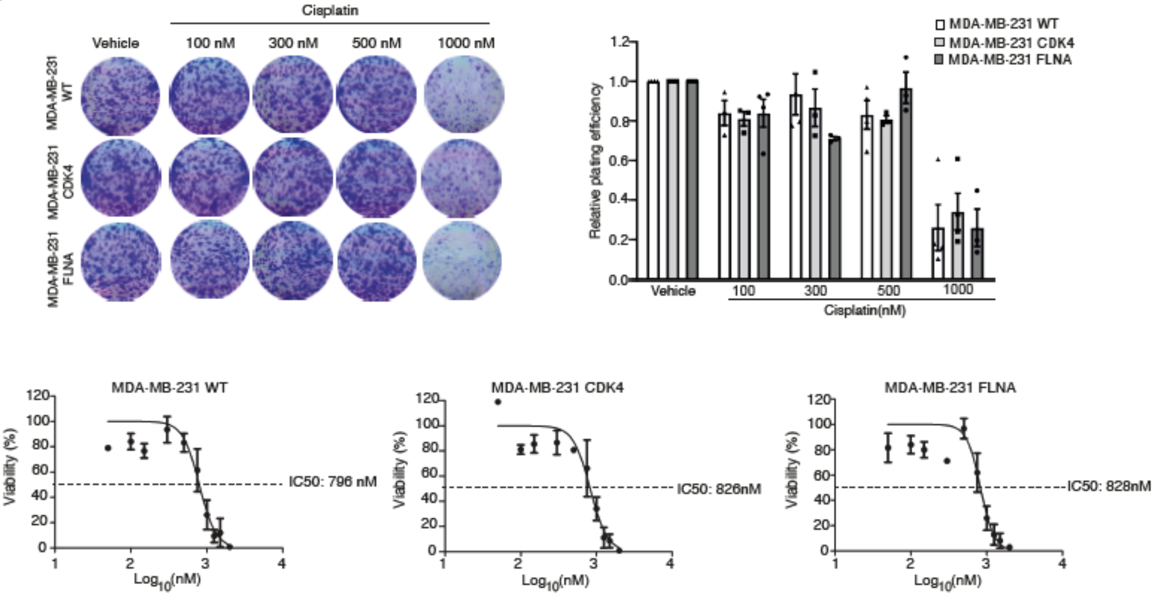

C

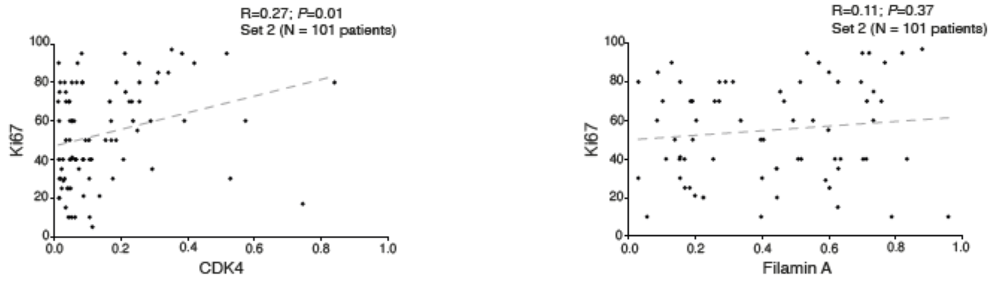

Supplementary Figure 9: Lack of sensitization to other cytotoxics by CDK4 or Filamin A overexpression. (A) Parental, CDK4- and Filamin A-overexpressing MDA-MB-231 cells were tested by colony assays for sensitivity to Adriamycin or **(B)** cisplatin. Data are represented as mean±SEM, n≥3 independent experiments; unpaired T-test. Neither the relative plating efficiency nor the IC50s (Mean±SEM, n≥3 independent experiments) showed meaningful differences between the parental cells and the CDK4 or Filamin A transfectants, suggesting that elevated CDK4 or Filamin induce only specific sensitization to paclitaxel. **(C)** This panel shows the correlation using a Pearson's correlation coefficient between CDK4 and Ki67 (left), and Filamin A and Ki67 (right) staining. Although the correlation between CDK4 and Ki67 was mildly positive (and expected), Filamin A did not show a statistically significant correlation with the replicative fraction. Source data are provided as a Source Data file.

Supplementary Figure 10

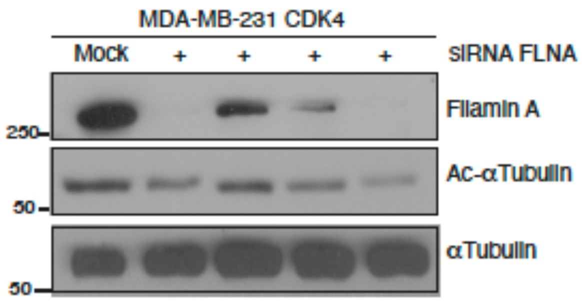

Supplementary Figure 10: Proportionate changes between Filamin A and acetylated alpha tubulin levels. Different experimental replicas where knock-down of Filamin A was attempted with siRNA and achieved variable efficiency (i.e., complete knockdown in lane 2, intermediate in lane 3, etc) showed a pairwise fluctuation of acetylated alpha tubulin levels along with those of Filamin A. Samples derive from the same experiment and blots were processed in parallel. Experiment was repeated twice (with 4 independent transfections each) with similar results. Source data are provided as a Source Data file.

Supplementary Figure 11

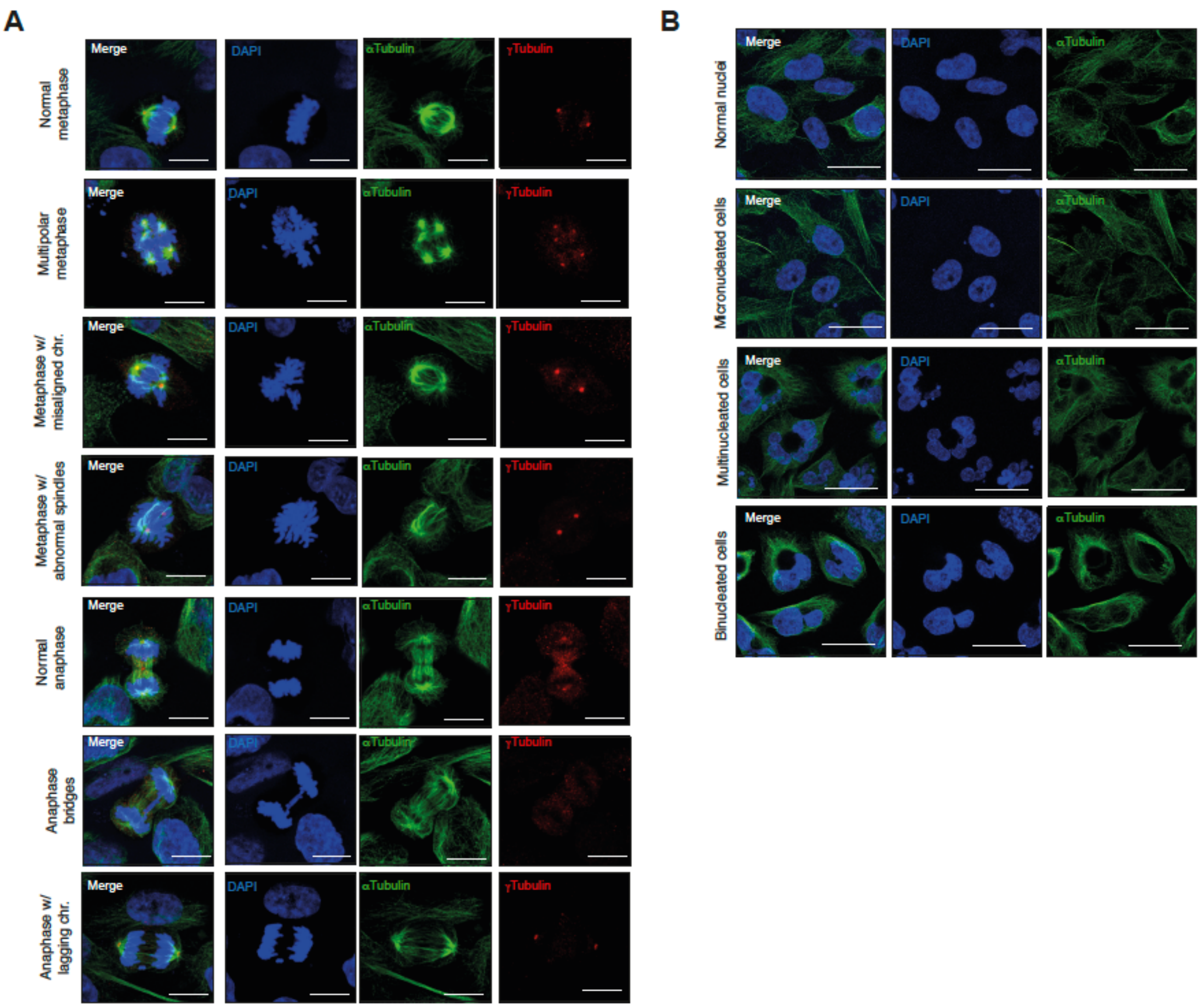

Supplementary Figure 11: Cell division aberrations pictures split by fluorescence channel. Confocal photomicrographs shown in Figure 7 are displayed here split by acquisition channel: from left to right, merged image, nuclear staining, alpha tubulin and gamma tubulin. Panel **(A)** shows the different found cell replication aberrations whereas panel **(B)** shows the observed nuclear aberrations resulting from the abnormal

replication process in response to paclitaxel. Scale bars: Panel A: 10 micrometers; Panel B: 25 micrometers.

Supplementary Tables

Supplementary Table 1: General clinical and demographic characteristics of the patients from the NCT01484080 trial that yielded valid samples for the study.

	Standard Arm (N=39)	Experimental Arm (N=46)	P value*
**Treatment**	Paclitaxel	Paclitaxel + nintedanib	N/A
**Age (median, range)**	48.8 (30.6 – 64.2)	47.0 (31.1 – 79.2)	0.816
**ECOG 0/1**	39 (100%)	46 (100%)	N/A
**Menopausal status**			0.280
Pre-menop.	20 (51.3%)	30 (65.2%)	
Menopausal	19 (48.7%)	16 (34.8%)	
**Hormonal receptors**			0.508
ER and/or PR +	29 (74.3%)	38 (82.6%)	
TNBC	10 (25.7%)	8 (17.4%)	
**Nodal status**			0.220
N0	19 (48.7%)	19 (41.3%)	
N1	20 (51.3%)	22 (47.8%)	
N2	0 (0%)	4 (8.7%)	
N3	0 (0%)	1 (2.2%)	
**Tumor size**			0.092
T1	0 (0%)	0 (0%)	
T2	32 (82.1%)	29 (63.0%)	
T3	7 (17.9%)	15 (32.6%)	
T4	0 (0%)	2 (4.4%)	
**Grade**			0.551
G1	7 (18.0%)	5 (10.9%)	
G2	19 (48.7%)	27 (58.7%)	
G3	13 (33.3%)	14 (30.4%)	
**Histologic sutype**			0.495
Ductal	31 (79.5%)	40 (86.9%)	
Lobular	7 (18.0%)	4 (8.7)	
Other	1 (2.5%)	2 (4.4%)	
**Ki67 (HR+ Only)**	N=29	N=38	0.827
14% or less	10 (34.5%)	11 (29.0%)	
>14%	19 (65.5%)	27 (71.0%)	
**Pathologic complete response to treatment**	4 (10.3%)	5 (10.8%)	1.000

*Age was compared with the Mann Whitney Wilcoxon test. Menopausal status, hormonal receptor status, Grade and Ki67 were compared with the Chi-Square test. Nodal status, tumor size, histologic subtype and pCR ratio were compared with the Fisher's test.

Supplementary Table 2: General mass spectrometry data description (total and by study arm)

	**Total*** **	**Experimental Arm Basal**	**Standard Arm Basal**
**MS/MS***	3075486	1025089	792680
**PSMs****	106012	34609	23135
**Phospho-PSMs**	55062	17761	11340
**% of enrichment**	51.94%	49.96%	45.93%
**Unique phospho-peptides**	3834	2174	1921
**Mono-phosphorylated**	3506	2020	1780
**Di-phosphorylated**	314	150	135
**Tri-/tetra-/-penta-phosphorylated**	14	4	6
**Phosphoproteins**	1352	1171	998
**Phosphosites**	3420	2531	2100
**Serine**	3005	2294	1921
**Threonine**	393	221	172
**Tyrosine**	22	16	7

*MS/MS: total detected mass spectra. **PSM: peptide-spectrum match.

***This table includes the information from the whole study, where the baseline samples from both treatment arms and the post-nintedanib samples from the experimental arm were run (first column). However, for the current manuscript, only the baseline samples

were processed; results regarding nintedanib effects on the proteome will be reported elsewhere. That is the reason why adding the numbers from the second and third columns (basal samples from the experimental and standard arms) does not add up the total numbers of PSMs, phospho-PSMs or other listed parameters.

Supplementary Table 3: Clinical characteristics of Set 1 patients.

Characteristic	Patients (N = 117)
**Age (median, range)**	48.5 (29 – 79)
**Subtype**	
ER and/or PR positive, HER2 non-amplified*	62 (53.0%)
HER2-amplified, any ER/PR	35 (30.0%)
ER, PR and HER2 negative	20 (17.0%)
**Tumor size**	
T1	31 (26.5%)
T2	64 (55.6%)
T3	17 (14.5%)
T4	4 (3.4%)
**Nodal stage**	
N0	50 (42.7%)
N1	51 (43.6%)
N2	13 (11.2%)
N3	3 (2.6%)
**Grade**	
G1	4 (3.4%)
G2	46 (39.3%)
G3	67 (57.3%)
**Neo-adjuvant chemotherapy****	117 (100%)
AC / P	70 (59.8%)
AC / P + a-HER2	35 (30.0%)
AC / PC	12 (10.2%)

*ER and/or PR positivity defined as staining of ER or PR in at least 5% of the tumor cells.

**Chemotherapy types: AC/P corresponds to 4 three-weekly courses of Adriamycin (60 mg/m2) and Cyclophosphamide (600 mg/m2) followed by 12 cycles of weekly Paclitaxel (80 mg/m2). The AC/P+a-HER2 regimen is identical to AC/P, but with the addition of

Trastuzumab 6 mg/kg on the first paclitaxel dose (loading dose) and Trastuzumab 2 mg/kg on weeks 2-12 and pertuzumab (840 mg on the first week of paclitaxel, and 420 mg on weeks 4, 7 and 10 of paclitaxel). Finally, AC/PC corresponds to 4 three-weekly courses of Adriamycin (60 mg/m2) and Cyclophosphamide (600 mg/m2) followed by 12 cycles of weekly Paclitaxel (80 mg/m2) plus Carboplatin (AUC = 2)

Supplementary Table 4: Clinical characteristics of Set 2 patients.

Characteristic	Patients (N = 101)
**Age (median, range)**	51.7 (33 – 87)
**Subtype**	
ER, PR and HER2 negative (TNBC)	101 (100%)
**Tumor size**	
T1	7 (6.9%)
T2	54 (53.5%)
T3	24 (23.8%)
T4	16 (15.8%)
**Nodal stage**	
N0	51 (50.5%)
N1	31 (30.7%)
N2	14 (13.9%)
N3	5 (4.9%)
**Grade**	
G1	1 (1.0%)
G2	21 (20.8%)
G3	67 (78.2%)
**Neo-adjuvant chemotherapy**	101 (100%)
AC / P*	101(100%)

* AC/P chemotherapy corresponds to 4 three-weekly courses of Adriamycin (60 mg/m2) and Cyclophosphamide (600 mg/m2) followed by 12 cycles of weekly Paclitaxel (80 mg/m2).

Supplementary Table 5: H-score cut-off points for the upper quartile of each validation antibody (Set 1: luminals, HER2 and TNBC cases)

Antibody	Q1 cutoff
**p-P70S6K (Thr^{389})**	H > 1.213
**p-Vim (Ser^{56})**	H > 0.580
**p-AMPK (Thr^{172})**	H > 1.226
**p-CAMK-IV (Tyr$^{196/200}$)**	H > 1.944
**p-Filamin A (Ser^{2152})**	H > 0.412
**CDK4**	H > 0.697
**Filamin A**	H > 0.612
**pan-p-PKC**	H > 0.750
**HMG-CoA Reductase Kinase**	H > 0.941
**p-YAP1 (ser^{127})**	H > 1.026
**Plectin**	H > 1.583

Supplementary Table 6: H-score cut-off points for the upper quartile of each validation antibody (Set 2: only TNBC cases)

Antibody	Q1 cutoff
**p-P70S6K (Thr^{389})**	H > 0.745
**p-Vim (Ser^{56})**	H > 0.611
**CDK4**	H > 0.406
**Filamin A**	H > 0.782
**HMGCOA Reductase Kinase**	H > 1.745

Supplementary Data 1. Enriched phospho-peptides IDs among the different compared conditions. (Table provided in a separate Excel file)

The table comprises 3 sheets, corresponding to the comparisons of responders versus non-responders in the standard arm, responders versus non-responders in the whole trial, and responders versus non-responders in the experimental arm (sheets 1, 2 and 3, respectively; each phospho-peptides list depicts the ID of the regulated phospho-peptides displayed in the volcano plots of Figures 3A, B, and C, respectively).

In each sheet, phospho-peptides are listed under a colored-heading depending on whether they belong to group 1 (red) or group 2 (orange) phospho-peptides regulated in each volcano plot.

For each peptide ID, the phosphorylated site and whether it corresponds to a serine (S), threonine (T) or Tyrosine (Y) is listed. The log-fold regulation in that condition (“Group”) as well as the *P* value are listed as well.

Supplementary Data 2 and 3: Mass spectrometry data of Tubulin and Filamin-A pull-downs. Supplementary Data 2 gathers Tubulin pull-down data comparing MDA-MB-231 WT and CDK4 cell lines. Supplementary Data 3 shows Filamin A pull down data in both cell lines (Tables provided in separate Excel files).

Supplementary Data 4: Percentage of cells displaying the observed mitotic (A) or nuclear (B) aberrations in response to paclitaxel or vehicle among the different transfectants: MDA-MB-231 WT, MDA-MB-231 CDK4 or MDA-MB-231 FLNA (Table provided in a separate Excel file).

Supplementary references

1. Alessi DR*, et al.* Mechanism of activation of protein kinase B by insulin and IGF-1. *EMBO J* **15**, 6541-6551 (1996).

2. Diehl JA. Cycling to cancer with cyclin D1. *Cancer Biol Ther* **1**, 226-231 (2002).

3. Oliva JL, Griner EM, Kazanietz MG. PKC isozymes and diacylglycerol-regulated proteins as effectors of growth factor receptors. *Growth Factors* **23**, 245-252 (2005).

4. Rasmussen H, Kojima I, Kojima K, Zawalich W, Apfeldorf W. Calcium as intracellular messenger: sensitivity modulation, C-kinase pathway, and sustained

cellular response. *Adv Cyclic Nucleotide Protein Phosphorylation Res* **18**, 159-193 (1984).

5. Beg ZH, Stonik JA, Brewer HB, Jr. Characterization and regulation of reductase kinase, a protein kinase that modulates the enzymic activity of 3-hydroxy-3-methylglutaryl-coenzyme A reductase. *Proc Natl Acad Sci U S A* **76**, 4375-4379 (1979).

Uncropped scans

Supplementary Figure 7A

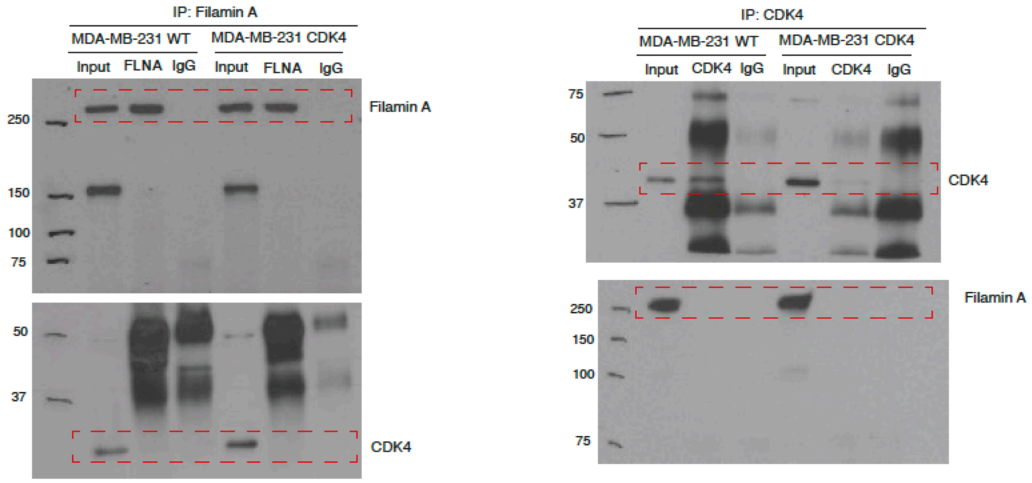

Supplementary Figure 10

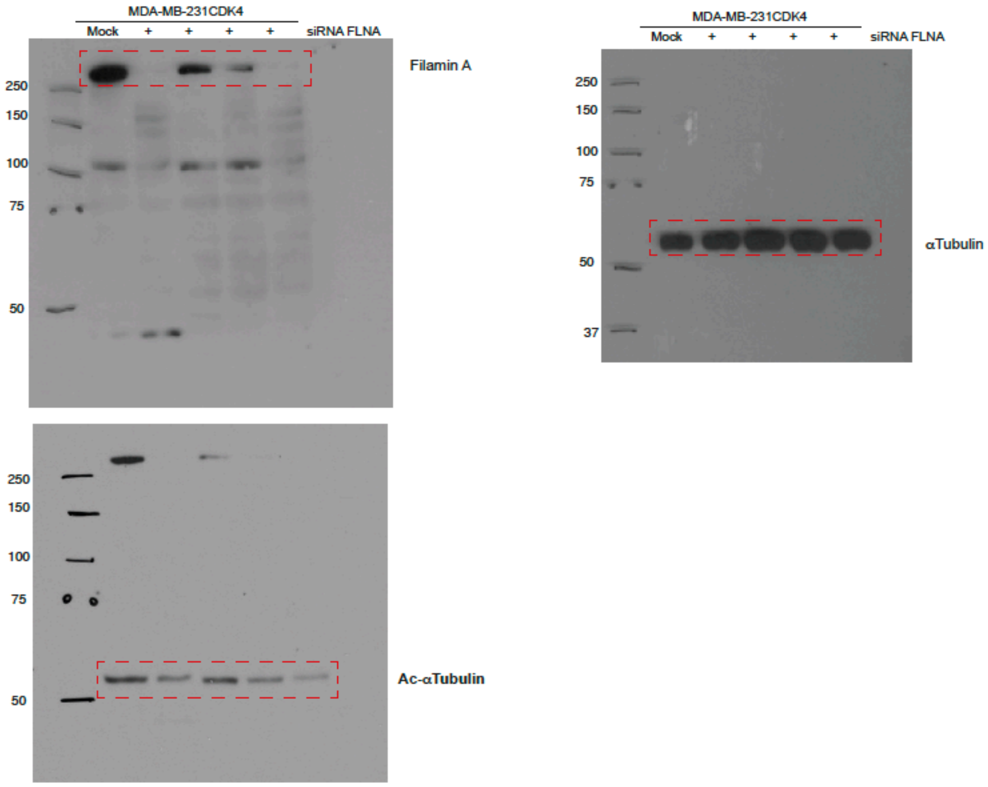

Supplement: Supplementary file 1 — Supplementary Information file [file 41467_2022_35065_MOESM1_ESM.pdf]
