# Supplementary material for: Phosphoproteomic analysis of neoadjuvant breast cancer suggests that increased sensitivity to paclitaxel is driven by CDK4 and filamin A
Source: Nat Commun. 2022 Dec 7;13:7529. doi: 10.1038/s41467-022-35065-z (PMC9729295; doi:10.1038/s41467-022-35065-z)
Supplement: Supplementary file 4 — Description of Additional Supplementary Files [file 41467_2022_35065_MOESM4_ESM.docx]

**Description of Additional Supplementary Files**

File Name: Supplementary Data 1

Description: Enriched phospho-peptides IDs among the different compared conditions

File Name: Supplementary Data 2

Description: Mass spectrometry data of Tubulin pull-downs

File Name: Supplementary Data 3

Description: Mass spectrometry data of Filamin-A pull-downs

File Name: Supplementary Data 4

Description: Percentage of cells displaying the observed mitotic (A) or nuclear (B) aberrations in response to paclitaxel or vehicle among the different transfectants: MDA-MB-231 WT, MDA-MB-231 CDK4 or MDA-MB-231 FLNA
